# Influence of Graphite/Graphene on the Tribological Behaviors of Self-Lubricating Fabric Composite

**DOI:** 10.3390/ma13010232

**Published:** 2020-01-05

**Authors:** Fang Ren, Sha Wang, Mingming Yu, Hongyu Duan, Meng Su, Musu Ren, Jinliang Sun

**Affiliations:** 1 Research Center of Composite Materials, School of Materials Science and Engineering, Shanghai University, Shanghai 200000, China; rfang@shu.edu.cn (F.R.); WangSha2015@163.com (S.W.); sumeng33@shu.edu.cn (M.S.); msren@shu.edu.cn (M.R.); jlsun@shu.edu.cn (J.S.); 2 Shanghai Bearing Technology Research Institute, Shanghai 200000, China; dhy8282@sina.com

**Keywords:** fabric composite, tribological, graphite/graphene, filler

## Abstract

Graphite/graphene particles were employed as functional fillers to modify hybrid polytetrafluoroethylene/polyisophthaloyl metaphenylene diamine (PTFE/Nomex) fabric-reinforced phenolic composites. The tribology behavior was investigated using a ball-on-disk wear tester, together with a 3D digital microscope. The graphite/graphene exhibits the synergetic effect from the results, which not only reduces the friction efficient but also improves the wear resistance of the composites. Moreover, the wear mechanisms were studied by the wear surface microstructure analysis. It is proposed that the synergetic effect includes mainly the positive rolling effect from the graphene and an improved load-carrying capacity brought by graphite. In addition, in order to obtain the optimized formulation to satisfy the bearing application, the influence of graphite and graphene content on the tribological property of the composites was studied in detail. On the basis of that, the application research was carried out on the bearing oscillating wear test, which will evaluate the engineering service life of the composite.

## 1. Introduction

Hybrid fabric composites composed of resin matrix and fibers have been extensively used in the fields of aviation and aerospace owing to their outstanding properties [1,2,3,4,5]. Among fabric fibers, polytetrafluoroethylene (PTFE) fibers display a low friction factor, and polyisophthaloyl metaphenylene diamine (Nomex) fibers possess the properties of high strength, high thermal stability, and good resistance to abrasion [6,7,8,9]. These excellent features make PTFE/Nomex fabrics good candidates to be used as tribomaterials. A typical structure for PTFE/Nomex fabric is featured with two distinct faces; one is rich in PTFE fibers for friction reduction, and the other is mainly Nomex fibers for supporting [6,10,11,12]. Traditionally, to achieve the tribopotential of fabric composites, phenolic resin is designed to combine with PTFE/Nomex fabrics owing to its good anti-wear and sufficient heat resistance properties [13,14,15]. However, the wear damage of PTFE/Nomex fabric composites can be easily accelerated by the easy loss of PTFE fibers and weak bonding between the resin matrix and fabric, influencing the service life of the composites [16,17,18,19]. Thus, it is critical to seek promotion on tribological properties for the PTFE/Nomex fabric composite, which is an essential part of the bearing [20,21,22].

Adding solid lubricant particles is demonstrated to be an effective method for improving the tribological properties of PTFE/Nomex fabric composites [23,24,25], especially for layered-structure particles such as graphite and graphene [26,27,28,29]. Generally, the reinforcement filler materials work together with the matrix in specific ways to realize different functional improvement for the fabric composites [30,31]. However, in regard to friction and wear properties, fillers that significantly enhance one property may not be equally efficient at improving the other. For example, Zhang et al. [32] investigated the tribological behaviors of 5–20 wt % graphite-filled PTFE/Nomex fabric composites. The study found that graphite fillers significantly increase the wear resistance (up to 20%) of the composites, but at the cost of a 2–10% increase in the friction coefficient. As the building block of graphite, in addition to the laminated structure, graphene particles display smaller particle size and higher thermal conductivity, enabling themselves as friction reduction agents. Ren et al. [27] studied the influence of graphene on the tribological properties of Nomex/phenolic composites. The study found that 6 wt % graphene provided around a 9% decrease in the friction coefficient, but resulted in a slight increase in the wear rate for the composite. In fact, researchers have found that it is increasingly hard to achieve the desired comprehensive tribological properties of fabric composite through only adding one type of reinforcement filler. Unfortunately, few studies have reported on the introduction of hybrid fillers into fabric composites. In view of this, combined fillers of graphite and graphene were considered to be incorporated into the PTFE/Nomex fabric composite, which is expected to complement the tribological behavior of the composite.

In this study, we introduced graphite/graphene to the phenolic resin to improve the tribological property of hybrid PTFE/Nomex fabric composites. The tribological behavior was investigated by the friction and wear tester and 3D digital microscope, based on which SEM was employed to study the wear mechanism of the composites. Furthermore, the optimal formulation of graphite/graphene fillers was discussed in detail. Finally, the modified composites were applied to spherical bearings, which were subjected to oscillating wear tests, and the service ability of the composite under actual working conditions was evaluated. This study is expected to improve the service life of fabric composites under low speed and overload conditions.

## 2. Experimental

### 2.1. Materials

**Figure 1:**
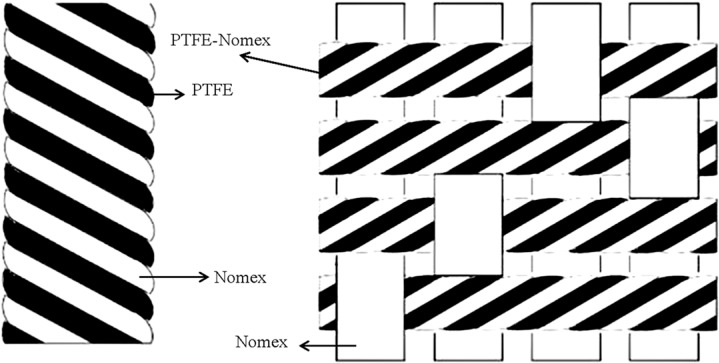
Weave structure of the polytetrafluoroethylene/polyisophthaloyl metaphenylene diamine (PTFE/Nomex) fabric.

The twill weave hybrid PTFE/Nomex fabrics used in this study were woven out of PTFE fibers (polytetrafluoroethylene, fineness: 400 DEN) and Nomex (polyisophthaloyl metaphenylene diamine, fineness: 200 DEN) provided by DuPont Plant. The weave structure and properties of the fabrics are shown in Figure 1 and Table 1, respectively. The adhesive resin (204 phenolic resin) was supplied by Shanghai XinHua Resin Co, Ltd., Shanghai, China. Micrographite (diameter: 48~75 μm) and multilayer graphene (diameter: 0.8~1 μm) were provided by the Sheng-quan Chemical Plant, China. The rest of the chemicals were all of analytical grade and used as received.

### 2.2. Specimen Preparation

**Figure 2:**
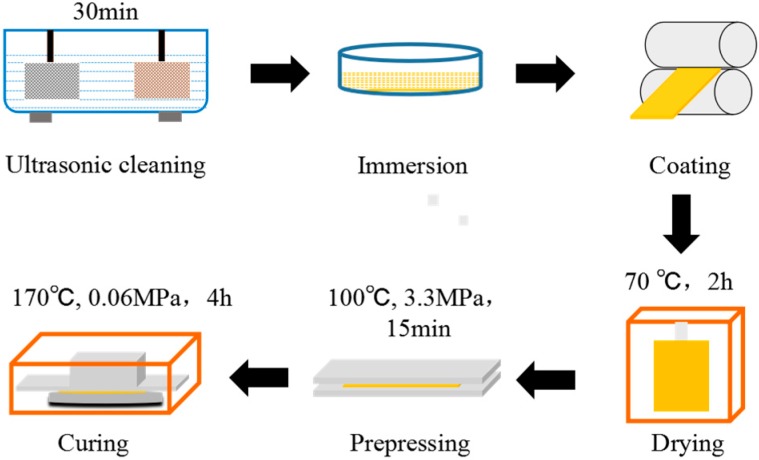
Preparation process of the hybrid PTFE/Nomex fabric composite.

Figure 2 shows the preparation process of the fabric composites; as is shown in the picture, the PTFE/Nomex fabrics were ultrasonically cleaned in ethanol for 30 min to remove surface impurities and dried in an oven at 70 °C for 20 min. Before immersion, the lubricant fillers were mixed uniformly with thermosetting phenolic adhesive resin at different mass fractions (shown in the composition of adhesive resin in Table 2) under high-speed mechanical stirring (5000 r/min, 10 min). Then, the hybrid PTFE/Nomex fabrics were immersed in the mixed or unfilled phenolic adhesive resin repetitively until the mass fraction of the fabric was about 75–80%. Afterwards, the immersed hybrid PTFE/Nomex fabrics were dried at 70 °C for 2 h and in an air atmosphere. After being dried, the fabrics were consolidated under 3–3.5 MPa at 100 °C. Finally, a series of unfilled and filled prepregs were affixed onto the AISI-1045 stainless steel (φ 43 mm × 3 mm, Ra: 0.45 μm) at 170 °C for 4 h under 0.02–0.08 MPa.

### 2.3. Tensile Strength Test

The tensile strength test of pure and graphite/graphene fillers-reinforced fabric composites were investigated by an MTS-CMT-4204 universal materials test machine (provided by MTS SYSTEMS Co, Ltd., Shanghai, China) by GB/T 33613-2017 standards at a constant speed of 20 mm/min. Each sample of tensile test was cut into 200 mm in length, 25 mm in width, and 400 ± 10 μm in thickness. The tensile strength of the composites was calculated through the formula σM = F (BD)^−1^, where F is the maximal pull force in N, B is the width in m, and D is the thickness in m.

### 2.4. Friction and Wear Test

**Figure 3:**
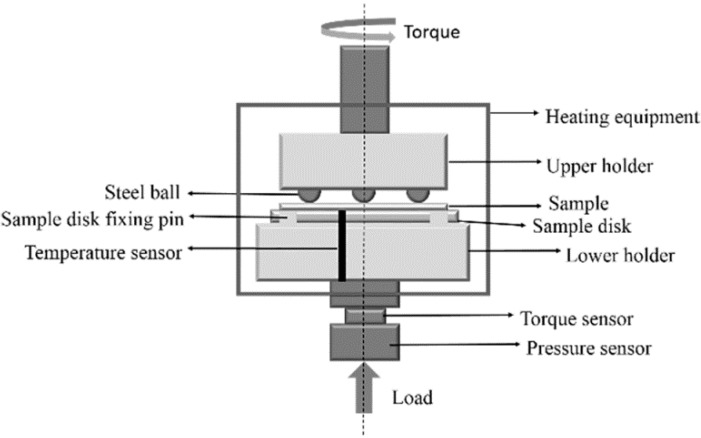
Schematic diagram of the ball-on-disk wear tester.

All friction and wear tests were performed in air atmosphere at room temperature and RH (relative humidity: 45–55%). The wear properties of hybrid PTFE/Nomex fabric composites were evaluated using a MMUD-5B ball-on-disk friction and wear tester (see Figure 3) provided by Jinan HengXu Testing Machine Technology Co., Ltd., Jinan, China. In the ball-on-disk tester, three AISI-1045 stainless steel balls (Ra: 0.15 μm, hardness: HRC50) were fixed equidistantly on the upper load arm with a chuck. During the test, the stationaryballs contact the flat sample disk that was affixed with composite specimen at 30 N and rotate on the liner with a rotating radius of 11 mm.

**Figure 4:**
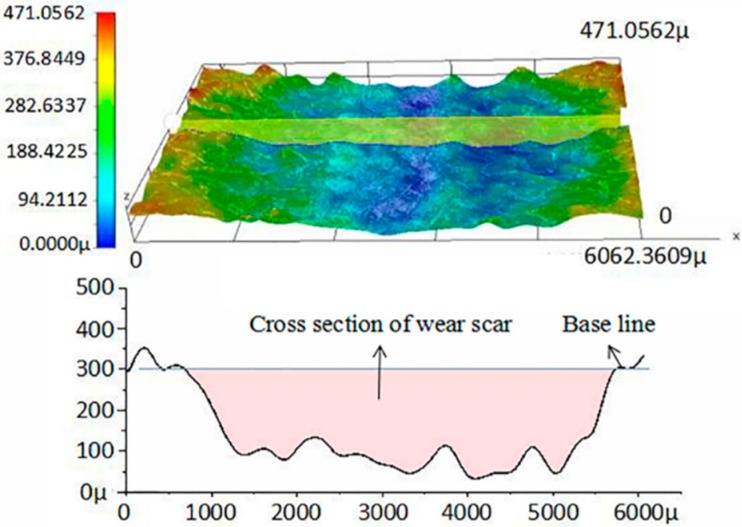
Schematic diagram of the measurement of the cross-sectional area and wear depth.

The dry sliding tests were performed on the testing machine at room temperature for 125 min, with the normal loads and sliding speed being 30 N and 0.115 m/s, respectively. At the end of each test, the wear volume loss (*V*) of the PTFE/Nomex fabric composites was obtained by measuring the depth and the cross-sectional area of the wear scar using the RH-2000 3D digital microscope, as show in Figure 4. The specific wear rate (ω, m^3^/N/m) of the composites is expressed by the equation ω = V/(P * L), in which V is the wear volume loss in m^3^, P is the load in N, and L is the sliding distance in m. We used the other wear rate in order to evaluate wear resistance more accurately. The formula of the time-related depth wear rate (*W_t_*) is as follows: *W_t_* = Δ*h/t*, in which t is the test time and Δ*h* is the height loss of the specimen. The running the friction-measure software was used to obtain the friction coefficient measured directly from the torque located under the sample disk. Each experiment was performed five times repeatedly, and the average values of the tests were reported.

### 2.5. Oscillating Wear Test

**Figure 5:**
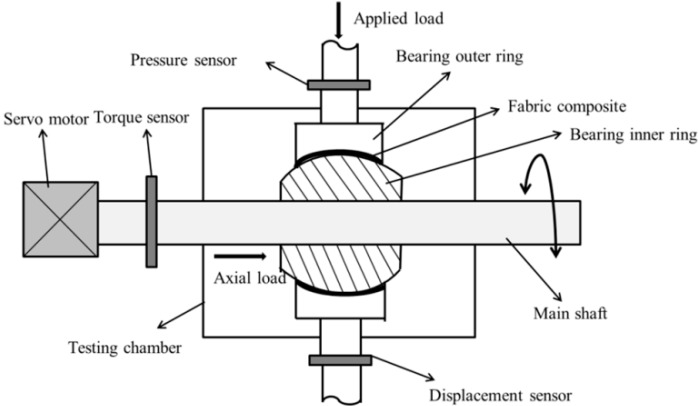
Schematic diagram of the oscillating wear tester.

The PLS-100 oscillating wear tester provided by Shanghai bearing technology research institute Co, Ltd., Shanghai, China was employed to investigate the oscillating wear behavior of the fabric composite under actual working conditions. On the tester (schematic diagram shown in Figure 5), a displacement sensor with a precision of 0.001 mm was fixed onto the load arm to obtain the wear loss data.

### 2.6. Characterization

An RH-2000 3D digital microscope (provided by Hirox China Co., Ltd., Shanghai, China) was firstly employed to acquire an accurate 3D surface profile of the worn surfaces; then, computerized image analysis software equipped on it was adopted to quantify surface parameters such as the depth and cross-sectional area of the wear scar. The morphologies of the worn surfaces of the composites and counterpart balls were analyzed by a Phenom scanning electron microscope (Shanghai, China with back scattered-electron mode at the accelerating voltage of 5 KV.

## 3. Result and Discussion

### 3.1. The Effect of Graphite/Graphene on the Tribological Property of Hybrid PTFE/Nomex Fabric Composite

**Figure 6:**
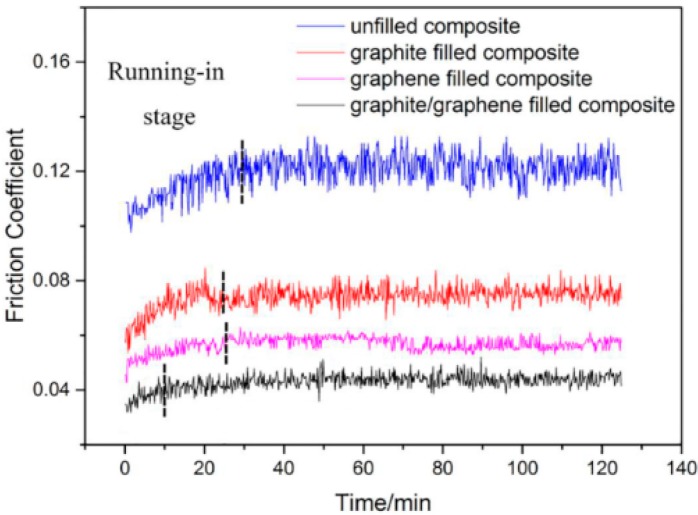
Friction coefficients of unfilled and a series of lubricant-filled PTFE/Nomex fabric composites.

**Figure 7:**
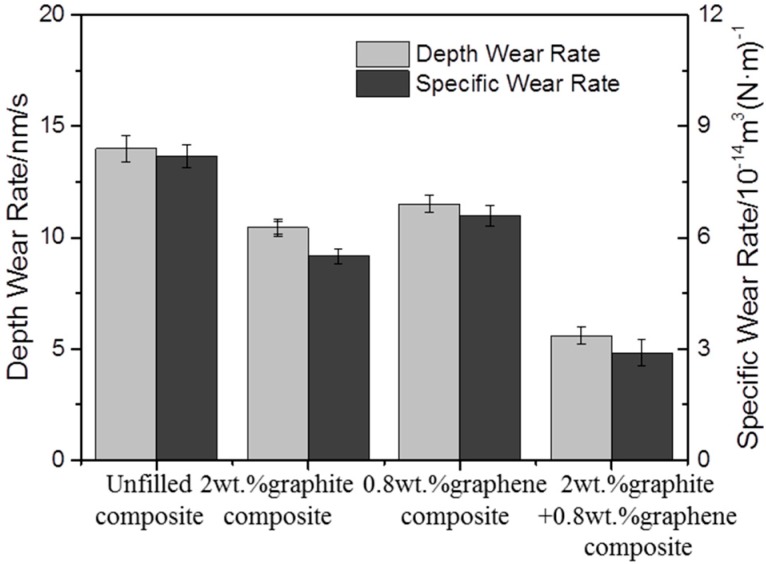
Wear rates of unfilled and a series of lubricant-filled PTFE/Nomex fabric composites.

**Figure 8:**
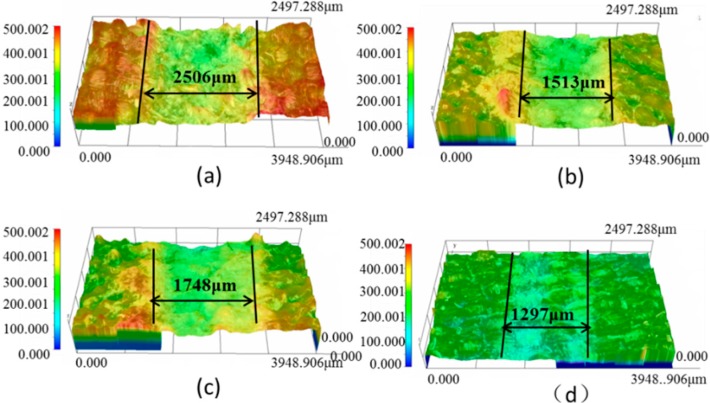
3D and profile of the worn surfaces for the hybrid PTFE/Nomex fabric composite: (**a**) pristine, (**b**) 2 wt % graphite, (**c**) 0.8 wt % graphene, (**d**) 2 wt % graphite + 0.8 wt % graphene.

Sliding wear tests were performed to investigate the effect of graphite/graphene on the friction and wear performance of PTFE/Nomex fabric composites, after which the friction curve and wear rate of the unfilled and different lubricant filled composite were given in Figure 6 and Figure 7, respectively. It indicated that the lubricant particle has a large influence on the friction and wear behaviors of fabric composites. Adding lubricant particles significantly decreased the friction coefficient and wear rate of the composite. Comparing the two particles, the graphite particle gives a greater wear resistance improvement, whereas the graphene particle is more efficient in the friction reduction of the composite. Most notably, the graphite/graphene filled PTFE/Nomex fabric composite showed a lower friction coefficient (μ: 0.042) and better anti-wear property (specific wear rate: 2.9 × 10^−14^ m^3^/(N·m), depth wear rate: 5.6 nm/s) than the graphite-filled and graphene-filled composites. The lowest friction coefficient was accompanied by the steady friction curve and a short running-in stage during the whole friction process, without compromising the friction reliability. The smallest wear rate is correspondent with the smoothest worn surface and narrowest wear scar expressed with the 3D microscopic images (see Figure 8).

**Figure 9:**
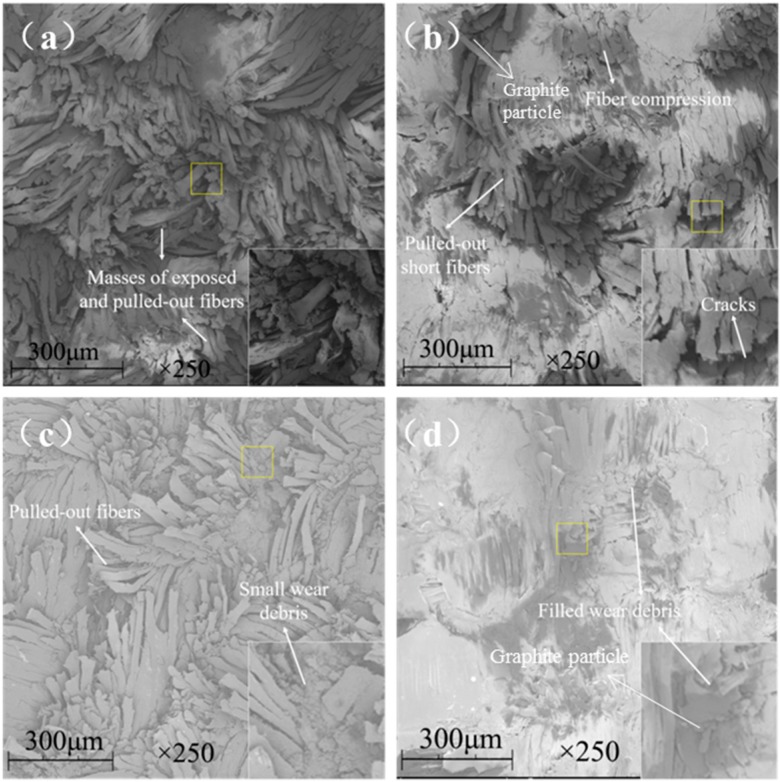
SEM images of the worn surfaces for the hybrid PTFE/Nomex fabric composites: (**a**) pristine, (**b**) 2 wt % graphite, (**c**) 0.2 wt % graphene, (**d**) 2 wt % graphite + 0.2 wt % graphene.

**Figure 10:**
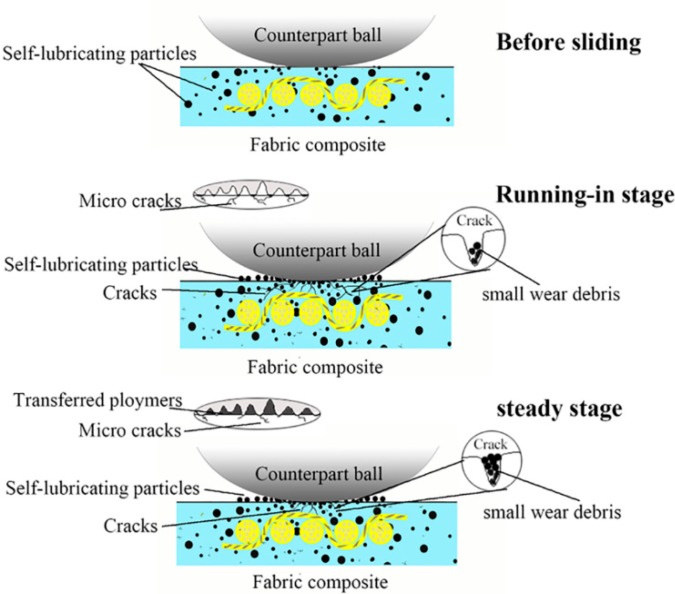
Wear model of the graphite/graphene-filled PTFE/Nomex fabric composite.

**Figure 11:**
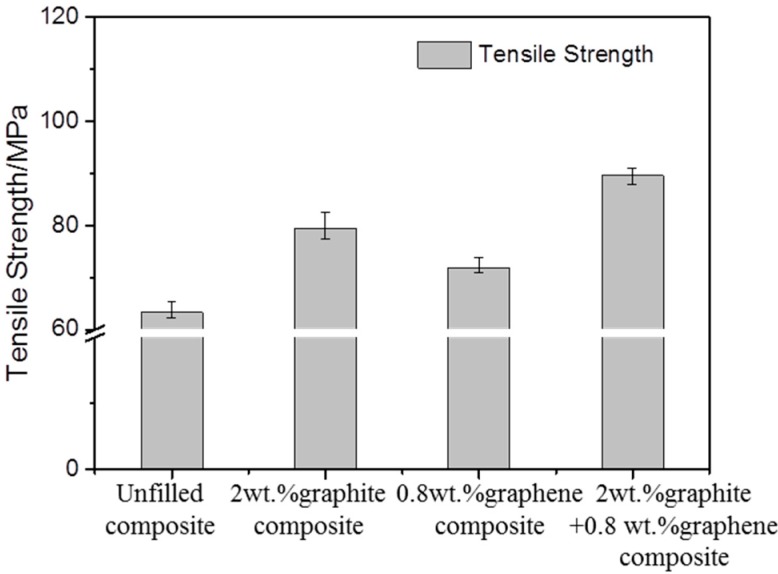
Strength test results of the unfilled and a series of lubricant-filled PTFE/Nomex fabric composites.

Figure 9 shows the SEM images of the worn surfaces of the unfilled, graphite-filled, graphene-filled, and graphite/graphene-filled PTFE/Nomex fabric composites. Significant wear occurred for the unfilled composite, as the most fiber exposure and fiber pullouts are observed on the worn surface. The wear of the graphite-filled composite is milder than the unfilled and graphene-filled composites. Some short and compressed fibers are exposed out of the worn surface that displays a layered structure with cracks, resulting from the improved load-carrying capacity brought by graphite particles. The graphene-filled composite displays many pulled-out fibers and much small wear debris on its worn surface. The tiny graphene particles may lead to a positive rolling effect to reduce the friction force; however, this effect results in abrasive wear that limits the improvement in wear reduction. For graphite/graphene-filled composites, it was found the mildest wear damage occurred on the worn surface, featuring few fiber pullouts, cracks, and almost no resin detachment on it. It is believed that the improved tribological property of the graphite/graphene-filled composite is attributed to the synergistic effect of graphite/graphene particles. Figure 10 shows the wear model of the graphite/graphene-filled composite. On one hand, tiny graphene particles lead to the positive rolling effect, which decreases the friction coefficient, during which small wear debris generated can repair the voids and holes to display the smoothest worn surface. On the other hand, the improved loading-carrying capacity brought by graphite particles significantly reduced the severe abrasive wear caused by graphene particles during the sliding process, leading, together with the groove-filling debris, to the mechanical property enhancement of the composite (see Figure 11).

### 3.2. The Effect of Graphene Content on the Tribological Property of the Graphite/Graphene-Filled PTFE/Nomex Fabric Composite

**Figure 12:**
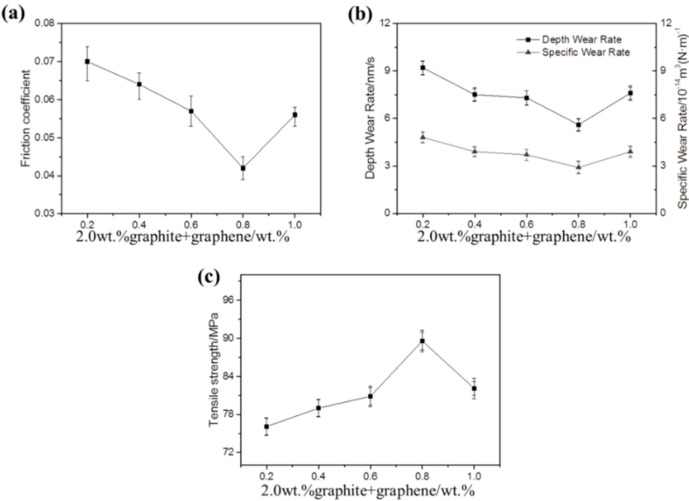
Friction coefficient (**a**) and specific wear rate or depth wear rate (**b**) of PTFE/Nomex fabric composites with different graphene contents; the tensile strength test results (**c**) of the corresponding fabric composites.

**Figure 13:**
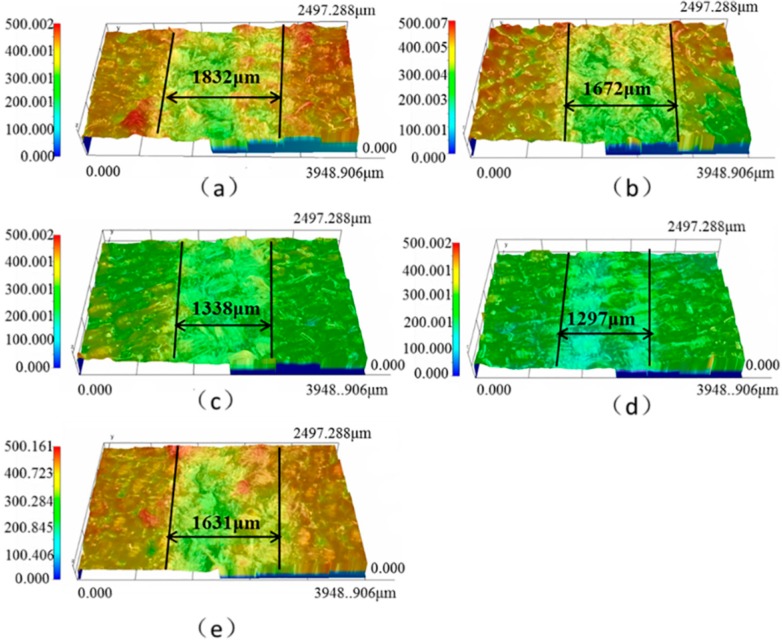
3D and profile of the worn surfaces for the filled hybrid PTFE/Nomex fabric composite: (**a**) 0.2 wt % graphene, (**b**) 0.4 wt % graphene, (**c**) 0.6 wt % graphene, (**d**) 0.8 wt % graphene, (**e**) 1.0 wt % graphene. (The content of graphite is a constant value of 2 wt %).

The friction coefficient, wear rate, and tensile strength of the different graphene content filled PTFE/Nomex fabric composite is shown in Figure 12a,b (the content of graphite is constant at 2 wt %). It can be seen that the graphene content has a large influence on the friction and wear property of the PTFE/Nomex fabric composite. Compared with the 0.2 wt % graphene filling, the friction coefficient and wear rate of the composite were reduced when 0.4 wt % graphene was filled, and when the content of graphene increased to 0.8 wt %, the graphite/graphene-filled composite exhibited the lowest friction coefficient and wear rate (μ: 0.042, specific wear rate: 2.9 × 10^−14^ m^3^/(N·m), depth wear rate: 5.6 nm/s). Figure 13 shows the 3D microscopic images of the worn surface of the composites. The composite filled with graphite/0.8 wt % graphene exhibited the smoothest worn surface and narrowest wear scar, corresponding to the lowest wear rate. However, further increasing the graphene content to 1 wt %, the friction coefficient and wear rate of the composite both increased, indicating a decrease in friction and wear property.

**Figure 14:**
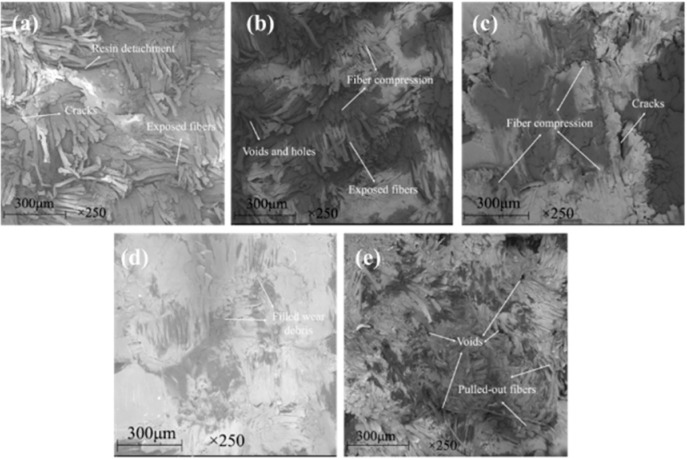
SEM images of the worn surfaces for the filled hybrid PTFE/Nomex fabric composite: (**a**) 0.2 wt % graphene, (**b**) 0.4 wt % graphene, (**c**) 0.6 wt % graphene, (**d**) 0.8 wt % graphene, (**e**) 1.0 wt % graphene. (The content of graphite is a constant value of 2 wt %).

**Figure 15:**
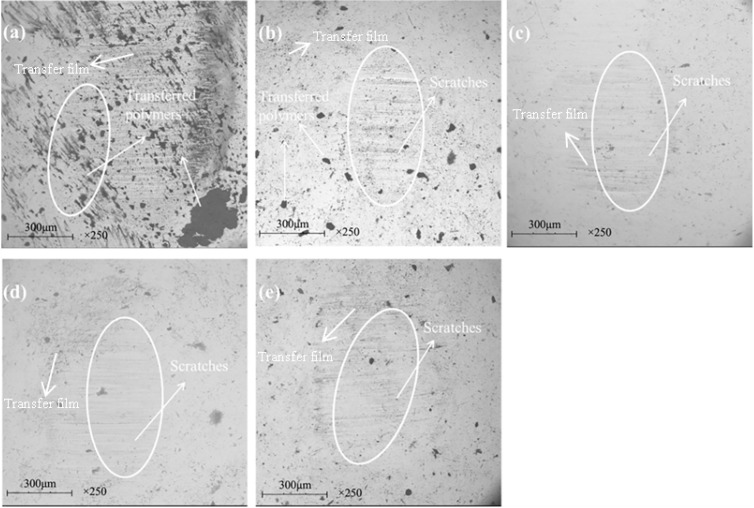
SEM images of the worn surfaces of the counterpart ball: (**a**) 0.2 wt % graphene, (**b**) 0.4 wt % graphene, (**c**) 0.6 wt % graphene, (**d**) 0.8 wt % graphene, (**e**) 1.0 wt % graphene. (The content of graphite is a constant value of 2 wt %).

Figure 14 and Figure 15 show the SEM images of the worn surface and its counterpart balls of the composites, respectively. When the graphene content is 0.2 wt %, resin detachment and fiber exposure were detected on the worn surface of the composite. With graphene content increasing, pulled-out fibers and adhesive resin tend to be compressed together to form a smoother worn surface that is featured with less holes and cracks. When the graphene content increased to 0.8 wt %, the composite underwent the mildest wear damage. It is proposed that the graphene content increasing leads to a strengthened rolling effect and abrasive wear for the composite, generating more small-sized wear debris. As more wear debris accumulated in the voids and cracks generated during the sliding process, the worn surface of the composite was polished and smoothed, which was evidenced by the milder scratches and less transferred polymers on the counterpart balls (see Figure 15a–d). As a result, the mechanical property of the composite was enhanced (see Figure 13c), leading to a decrease in the wear rate. However, further increasing the graphene content caused more pulled-out fibers and voids on the worn surface (see Figure 14e) and more scratches and transferred polymers on the counterpart balls (see Figure 15e). It is believed that the overabundant graphene particles result in severe abrasive wear, causing a decrease in the mechanical (see Figure 12c) and anti-wear property of the composite.

### 3.3. The Effect of Graphite Content on the Tribological Property of the Graphite/Graphene-Filled PTFE/Nomex Fabric Composite

**Figure 16:**
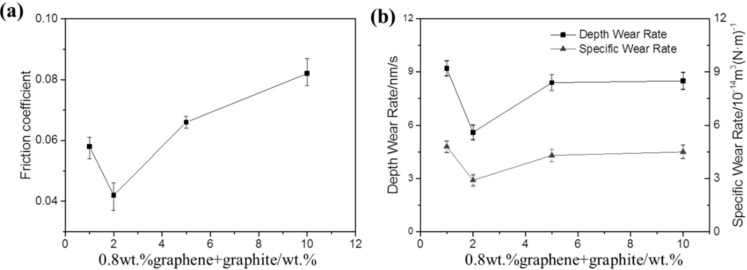
Friction coefficient (**a**) and specific wear rate or depth wear rate (**b**) of PTFE/Nomex fabric composites with different graphite contents.

**Figure 17:**
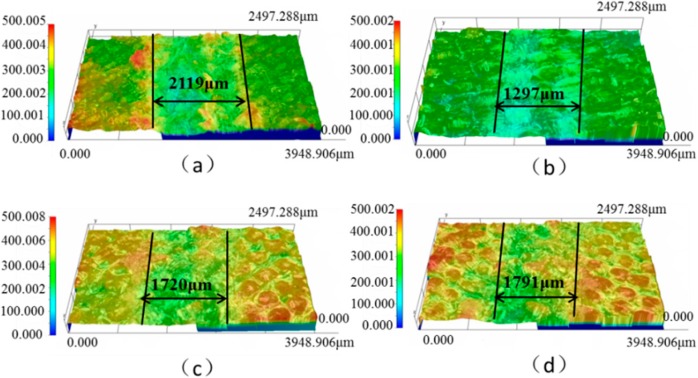
3D and profile of the worn surfaces for the filled hybrid PTFE/Nomex fabric composite: (**a**) 1 wt % graphite, (**b**) 2 wt % graphite, (**c**) 5 wt % graphite, (**d**) 10 wt % graphite. (The content of graphene is a constant value of 0.8 wt %).

The effect of graphite content on the friction coefficient and wear rate of the graphite/graphene-filled PTFE/Nomex fabric composite is shown in Figure 16 (the content of graphene is constant at 0.8 wt %). It is seen that the friction coefficient and wear rate decrease significantly with the content of graphite content increasing up to 2 wt %. However, with a further increase of graphite content, the friction coefficient and wear rate increased, indicating the decreased friction and wear property of the composite. The 3D microscopic images (see Figure 17) of the worn surface of the composites show that all the composites exhibit a broader wear scar and rougher worn surface than the composite filled with 2 wt % graphite, corresponding to the friction and wear results.

**Figure 18:**
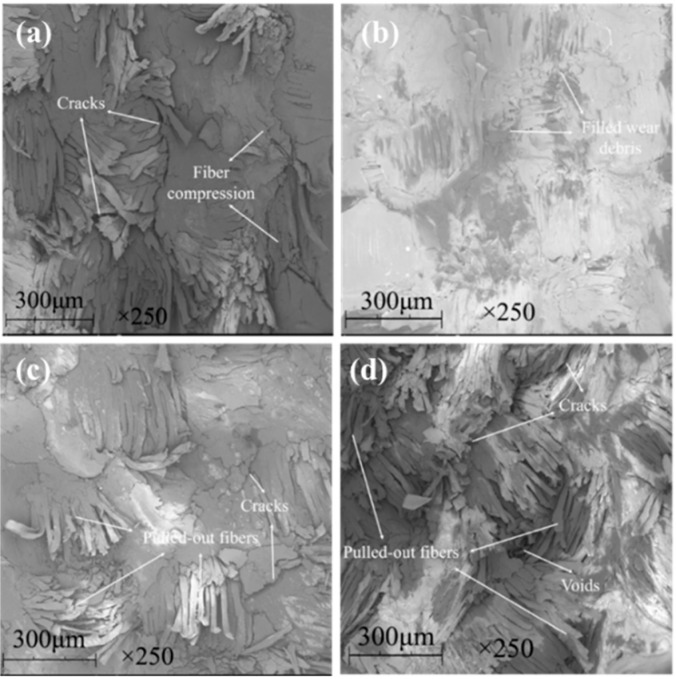
SEM images of the worn surfaces for the filled hybrid PTFE/Nomex fabric composite: (**a**) 1 wt % graphite, (**b**) 2 wt % graphite, (**c**) 5 wt % graphite, (**d**) 10 wt % graphite. (The content of graphene is a constant value of 0.8 wt %).

**Figure 19:**
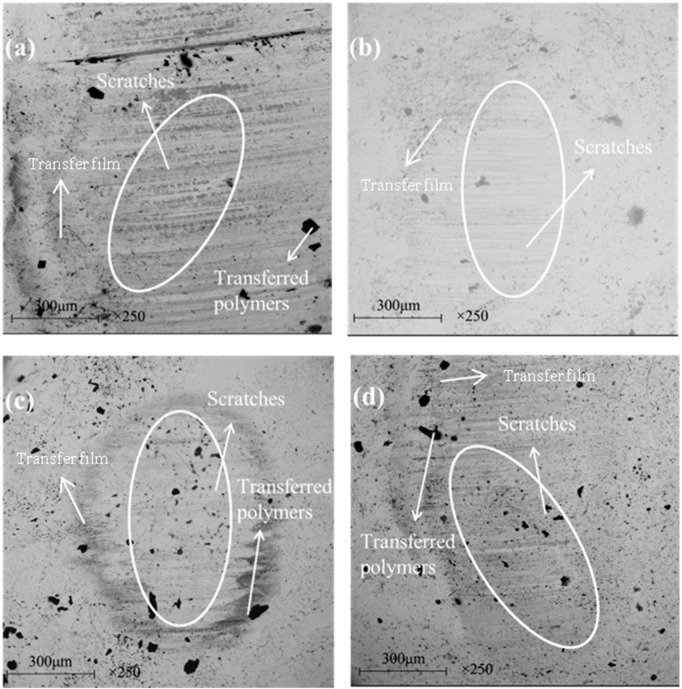
SEM images of the worn surfaces for the filled hybrid PTFE/Nomex fabric composite: (**a**) 1 wt % graphite, (**b**) 2 wt % graphite, (**c**) 5 wt % graphite, (**d**) 10 wt % graphite. (The content of graphene is a constant value of 0.8 wt %).

Figure 18 and Figure 19 show the SEM images of the worn surface and its counterpart balls of the composites. When the graphite content is 1 wt %, the worn surface exhibited a layered structure with compressed fibers and cracks (see Figure 18a). The worn surface of the counterpart ball showed numerous scratches and a large area of transferred polymers, indicating a relatively serious wear damage (see Figure 19a). With the graphite content increasing up to 2 wt %, the exposure of fibers was scarce (see Figure 18b), and the counterpart ball was better protected, as less scratches and transferred polymers were observed on it (see Figure 19b). It is proposed that with the graphite content increasing, the strengthened effect of the interlayer slip of multilayer structured graphite improves the load-carrying capacity of the composite, leading to milder wear damage. However, after further increasing the graphite content, the worn surface of the composites exhibited masses of pulled-out fibers and cracks, resulting in an increase in surface roughness. It is believed that the overabundant graphite fillers result in a decrease in the continuity of the resin matrix, causing the increased detachment of the resin matrix and wear debris (see Figure 18c,d), as more scratches are observed on the worn surfaces of the counterpart balls (see Figure 19c,d).

### 3.4. Oscillating Wear Test for Bearing Application

#### 3.4.1. Experimental

As the PTFE/Nomex fabric composite filled with 2 wt % graphite and 0.8 wt % graphene demonstrated the most favorable tribological property, the composite was used on the spherical bearing, which was then subjected to an oscillating wear test. The AISI-1045 bearing used consisted of an inner ring (diameter: 15 mm) for friction and an outer ring (diameter: 28 mm) affixed with the fabric composite.

When prepare the bearing, the composite prepreg (which was composed of the PTFE/Nomex fabric, the phenolic resin, and the graphite/graphene fillers) was firstly affixed onto the internal surface of the bearing outer ring, and then cured with a certain pressure; after that, the bearing sample was prepared.

The oscillating wear test was performed under actual working conditions, with the testing parameters shown in Table 3.

#### 3.4.2. Wear Test Results

**Figure 20:**
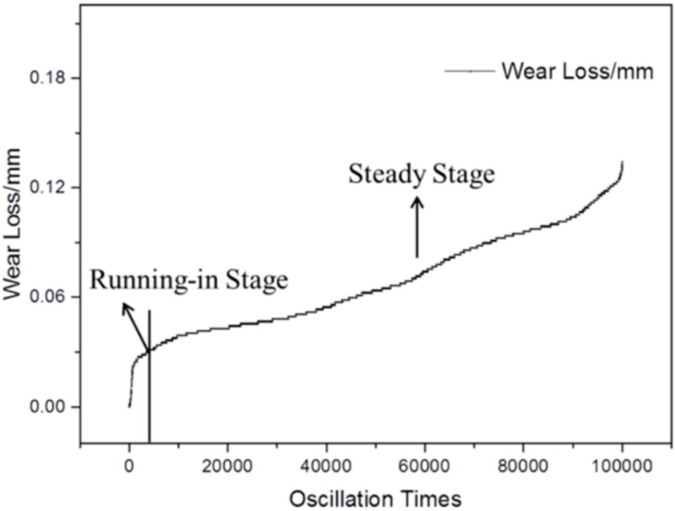
The wear loss changes of the composite as a function of oscillation times.

To further evaluate the engineering service life of the modified composite, oscillating wear tests were performed for the composite under actual working conditions. Figure 20 illustrated the wear loss changes of the composite as a function of oscillation times. It can be seen that the whole wear process was divided into two stages. After a short running-in stage, the wear of the composite quickly entered into a steady stage in which the wear loss linearly increases with the oscillation times. Based on the actual application conditions, the wear loss of the existing limited market bearing products is about 0.19 mm. After 100,000 oscillations, the wear loss was calculated at 0.134 mm, which indicates the improved anti-wear property for fabric composites used in spherical bearings.

#### 3.4.3. Worn Surface Analysis

**Figure 21:**
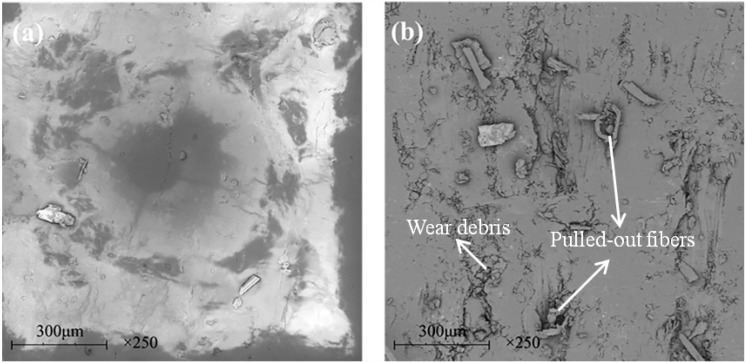
SEM images of the modified composite surface before and after test. (**a**) Original surface, (**b**) worn surface after 100,000 oscillations.

Figure 21 shows the SEM images of the composite surfaces before and after wear tests. It can be seen that after 100,000 oscillations, the worn surface of the composite exhibited few pulled-out fibers and wear debris. Besides, the resin matrix rarely detached from the fabric, indicating a mild wear damage. The results indicate that the composite filled with 2 wt % graphite/0.8 wt % graphene meets the requirement from actual working conditions.

## 4. Conclusions

Graphite/graphene particles were employed as hybrid fillers to modify hybrid PTFE/Nomex fabric-reinforced phenolic composites. The tribological property and oscillating wear behavior of the modified composites were studied comprehensively. The results show that the hybrid graphite/graphene fillers achieve a comprehensive improvement in friction and wear properties for PTFE/Nomex fabric composites. SEM and 3D digital microscope analysis reveal that the improvement is mainly attributed to the positive rolling effect of graphene particles and load-carrying capacity enhancement brought by graphite particles, which is illustrated in the wear model for the composite. Oscillating wear tests under actual working conditions were performed to evaluate the service ability of graphite/graphene-filled composites, which demonstrates the advantage of hybrid fillers on engineering service life promotion and bearing application as reinforcements.

## Figures and Tables

**Table 1:** Properties of the PTFE/Nomex fabric.

	Weave Density (Threads/Inch)		Filament Numbers of a Yarn	
Fabric	Nomex-PTFE	Nomex	PTFE	Nomex
PTFE/Nomex	35	55	60	100

**Table 2:** Composition of adhesive resin.

Final Sample	Phenolic Resin (wt %)	Graphite (wt %)	Grapheme (wt %)
1#	100		
2#	97.8	2	0.2
2#	97.6	2	0.4
4#	97.4	2	0.6
5#	97.2	2	0.8
5#	97	2	1.0
6#	98.2	1	0.8
7#	94.2	5	0.8
8#	89.2	10	0.8

**Table 3:** Parameters of oscillating wear test.

Applied Load	Frequency	Oscillation Angle	Temperature	Oscillation Times
44.4 kN	0.2 Hz	±25°	25 °C	100,000
